# A case of lipoprotein glomerulopathy with thrombotic microangiopathy due to malignant hypertension

**DOI:** 10.1186/1471-2369-14-53

**Published:** 2013-02-28

**Authors:** Yu Wu, Xiaohan Chen, Yuan Yang, Baohe Wang, Xiaoxia Liu, Ye Tao, Ping Fu, Zhangxue Hu

**Affiliations:** 1 Department of Hematology, West China Hospital, National Key Laboratory of Biotherapy of Human Diseases, Sichuan University, Chengdu, Sichuan Province, China; 2 Department of Nephrology, West China Hospital, National Key Laboratory of Biotherapy of Human Diseases, Sichuan University, Chengdu, Sichuan Province, China; 3 Department of Medical Genetics, West China Hospital, National Key Laboratory of Biotherapy of Human Diseases, Sichuan University, Chengdu, Sichuan Province, China; 4 Department of Nephrology, Nuclear industry 416 Hospital, Chengdu, Sichuan Province, China

**Keywords:** Lipoprotein glomerulopathy, Thrombotic microangiopathy, Malignant hypertension, *APOE* Kyoto

## Abstract

**Background:**

Lipoprotein glomerulopathy (LPG) is a rare inherited renal disease characterized by intraglomerular lipoprotein within the lumina of severely dilated glomerular capillaries. The common clinical presentation of LPG includes proteinuria or nephrotic syndrome. Hypertension and anemia were thought to be mild in LPG. Thrombotic microangiopathy (TMA) in LPG has not been previously reported. In this report, we present a patient with LPG that developed TMA. To the best of our knowledge, this is the first report of TMA in LPG.

**Case presentation:**

Four years ago (2005), a 19-year-old Chinese woman was diagnosed with nephrotic syndrome and provided prednisone treatment. A combination of prednisone and cyclophosphamide did not have any effect and was discontinued after six months. Although she was steroid-resistant, over the next subsequent three years, she maintained normal renal function without anemia and thrombocytopenia. In February 2009, she had a severe headache and blurry vision and presented at a local hospital with severe hypertension. Blood pressure was 220/160 mmHg. Laboratory data showed hemoglobin 3.8 g/dL; platelet counts 29×10^9^/L; urinary protein 7.90 g/d; total bilirubin 29.9 umol/L; indirect bilirubin 28.2 umol/L; LDH 1172 U/L; ALB 2.66 g/dL; urea nitrogen 52 mg/dL; serum creatinine 3.2 mg/dL; triglyceride 253 mg/dL; total cholesterol 273 mg/dL. ANA, ds-DNA, ANCA, anti-GBM antibody and anticardiolipin were all negative. A renal biopsy revealed LPG with TMA. Genetic evaluation showed the patient carried the *APOE* Kyoto mutation. Adequate control of blood pressure improved microangiopathic anemia and thrombocytopenia, however, renal function did not improve and she eventually developed uremia and became hemodialysis dependent.

**Conclusion:**

We report on a rare case of TMA probably due to malignant hypertension in LPG. Early lipid-lowering and antihypertensive treatment may improve outcome. The pathophysiologic relationship between LPG and TMA should be investigated further.

## Background

Lipoprotein glomerulopathy (LPG) is a rare inherited renal disease characterized by the presence of intraglomerular lipoprotein thrombi due to deposition of lipid droplets within the lumina of severely dilated glomerular capillaries, with a variable degree of mesangial proliferation and segmental glomerular sclerosis [1,2]. The common clinical presentation of LPG includes proteinuria or nephrotic syndrome, which may gradually progress to chronic renal failure. Despite deposition of numerous intraglomerular lipoprotein thrombi, no thrombi in arterioles have been described in LPG patients. Moreover, acute renal failure has rarely developed in LPG patients. In the report herein, we present a case of LPG with thrombotic microangiopathy (TMA). The relationship between LPG and TMA is discussed.

## Case presentation

A 19-year-old Chinese woman was admitted to our hospital in April 2009. Four years prior to this admission, she noticed mild pretibial edema. At that time, urinalysis showed proteinuria 3+. Blood pressure was normal. She was diagnosed with nephrotic syndrome. Oral prednisone was initiated for 2 months with a dosage of 1 mg/kg per day, then tapered. The total period of prednisone treatment was 6 months. During this period, cyclophosphamide 100 mg per day orally was added for 10 days. The total cyclophosphamide dosage was 1 g. The patient did not improve with this regimen. During the next 3 years (2005–2008), she maintained stable renal function and normal blood pressure. Six months before admission (November 2008), hypertension developed at 150/90 mmHg. The 24-hour urinary protein was 8.8 g/d. Laboratory findings showed a serum total protein of 4.23 g/dL, serum albumin 2.46 g/dL, triglyceride 241 mg/dL, total cholesterol 350 mg/dL, HDL-C 71 mg/dL, and LDL-C 233 mg/dL. A complete blood count showed a hemoglobin of 9.8 g/dL, WBC counts 6.6×10^9^/L, and platelet counts 130×10^9^/L. Felodipine 5 mg per day was administered to keep blood pressure at a normal range. Just two months before admission (February 2009), she felt a severe headache, blurry vision, nausea, vomiting, and presented to a local hospital. On admission at that hospital, physical examination showed blood pressure was up to 220/160 mmHg, with facial pallor, and pitting edema of the lower extremities. Laboratory data showed a hemoglobin of 3.8 g/dL; WBC counts 3.75 × 10^9^/L; platelet counts 29 × 10^9^/L; urinary protein 7.90 g/d; total bilirubin 29.9 umol/L; indirect bilirubin 28.2 umol/L; ALT 22 U/L; AST 36 U/L; LDH 1172 U/L; ALB 2.66 g/dL; urea nitrogen 52 mg/dL; serum creatinine 3.2 mg/dL; triglyceride 253 mg/dL; total cholesterol 273 mg/dL, HDL-C 46 mg/dL, LDL-C 200 mg/dL, VLDL-C 50 mg/dL, apoA1 0.90 mg/dL, apoB 1.21 mg/dL. ANA, ds-DNA, ANCA, anti-GBM antibody and anticardiolipin were all negative. HIV, HBV and HCV tests were all negative. Urine HCG was negative. A brain CT scan did not reveal any abnormality. The patient did not have any evidence of malignancy, pregnancy or abortion. During this period, she had not experienced diarrhea or infections. There was no family history of renal diseases, hypertension or hematologic diseases.

**Figure 1:**
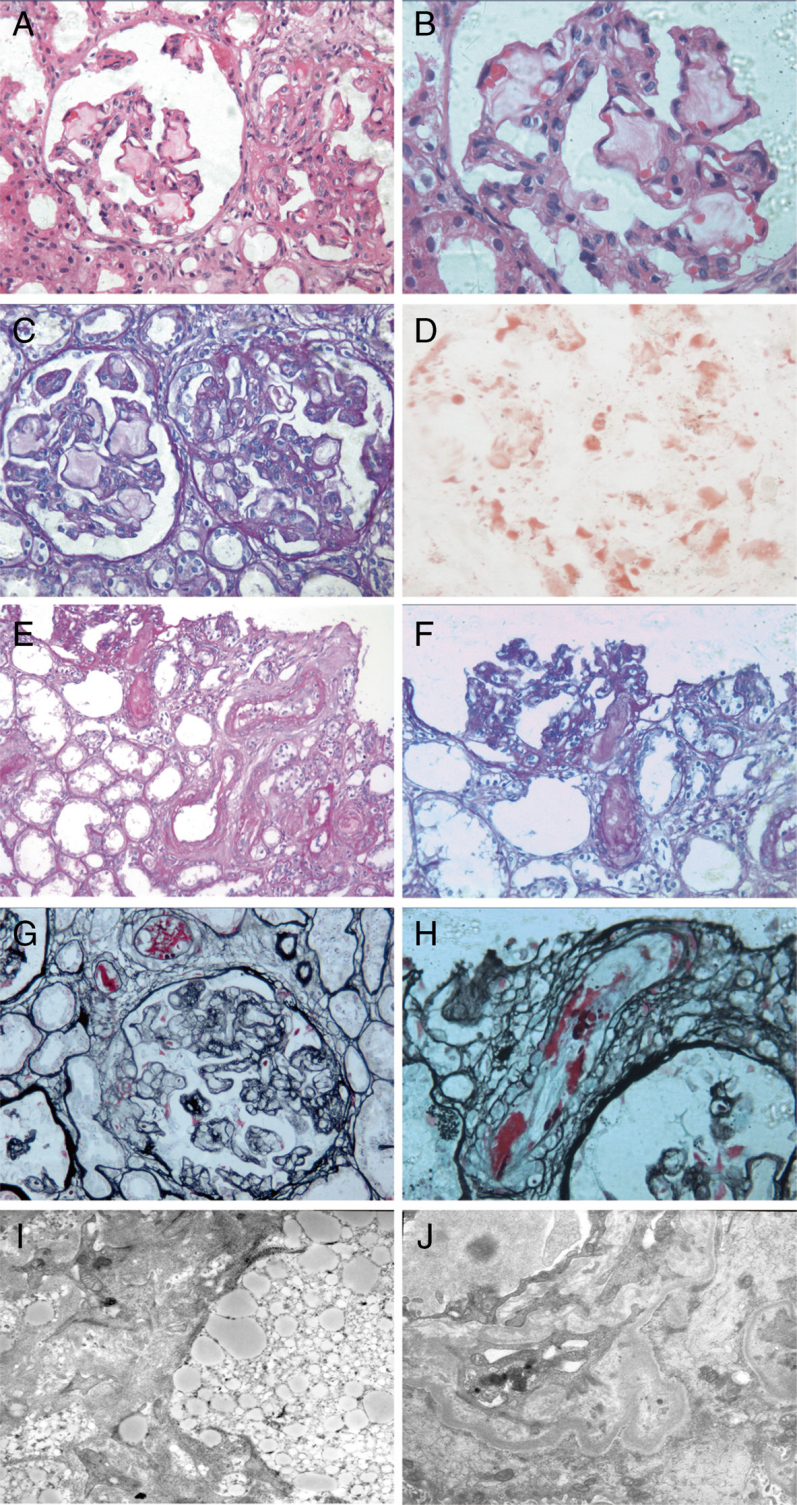
**Glomerular and vascular findings from renal biopsy specimens in a patient with LPG and TMA. **Panel **A **shows a glomerulus with the presence of intraglomerular lipoprotein thrombi due to deposition of lipid droplets within the diluted lumina of glomerular capillaries. Although lipoprotein thrombi were present in glomerular capillaries, the glomerulus still exhibited ischemic features (HE staining; ×200). In Panel **B**, the glomerulus shown in Panel **A **were enlarged (HE staining; ×400). In Panel **C**, glomeruli shown in Panel **A **were stained in PAS, also showing intraglomerular lipoprotein thrombi and ischemic features (PAS staining; ×200). In Panel **D**, lipid was stained positive (Oil Red O staining; ×400). In Panel **E**, a thrombus in the lumina of arteriole which extended to the vascular pole of the adjacent ischemic glomerulus is shown. Other arterioles also showed thrombus and pronounced swelling of endothelial cells (PAS staining; ×200). In Panel **F**, the thrombus in the arteriole extended to the vascular pole of an adjacent ischemic glomerulus shown in Panel **E** was enlarged (PAS staining; ×400). In Panel **G**, a glomerulus was shown with an extensive double-contour formation. Few red blood cells are observed in the capillaries. Adjacent arterioles with a swelling of endothelial cells and the presence of a fluffy material (fibrin) that caused a narrowing of vascular lumen are shown (PASM staining; ×400). In Panel **H**, endothelial swelling with mucoid change and chunks of fibrin in interlobular arteries are shown. In Panel **I**, the capillary lumen were occluded with various granule sizes. In Panel **J**, the subendothelial zone was focally expanded by electron-lucent material, and a new glomerular basement membrane was formed. Panels **I** &**J** are electron microscopy images.

A kidney biopsy was performed. Light and electron microscopic examinations of renal biopsy material showed a marked dilatation of the capillary lumen in the glomeruli, and the presence of laminated intraglomerular lipoprotein thrombi due to deposition of lipid droplets within the glomerular capillaries (Figure 1A,B,C). Immunofluorescence showed IgG, IgA, IgM, C3, C4, C1q were all negative. Oil Red O staining was positive (Figure 1D). Electron microscopy showed that the glomerular capillary lumina were occupied by granules of various sizes (Figure 1I). She was diagnosed with LPG and treated with antihypertensive drugs (Nifidipine 60 mg + Prazosin 12 mg + Metoprolol 50 mg per day) and Simvastatin. At the same time, she received hemodialysis and red blood cell transfusion. Over the next four weeks, blood pressure was observed to be under control (average level 135/80 mmHg). Anemia and thrombocytopenia were improved. Hemoglobin was 7.8 g/dL, platelet counts were 110 × 10^9^/L; albumin also increased to 3.17 g/dL; but renal insufficiency was aggravated, with urea nitrogen 24 mg/dL, and serum creatinine 7.2 mg/dL. Urine output decreased progressively to anuria. The patient was then transferred to our hospital.

Because LPG has rarely developed into irreversible acute renal failure, a re-examination of the renal biopsy specimens was performed. Among the 17 glomeruli sampled for light microscopy, three were globally sclerotic, and one was segmental sclerotic. The size of the glomeruli varied greatly. The seven greatly-enlarged glomeruli showed severe dilated capillaries filled with a foamy, whorled, mesh-like material in the glomerular capillary lumens. The other six moderately-enlarged glomeruli showed extensive ischemic changes, retraction of the glomerular tuft and marked thickening and wrinkling of the capillary wall (Figure 1A,B,C). Even in the ischemic glomeruli, a thrombus-like material was observed in some capillaries, so the sizes of these glomeruli were still larger than normal glomeruli, which made the ischemic appearance of these glomeruli hard to identify. Double-contour formations were obvious. Vascular lesions were severe. Swelling of endothelial cells and fluffy masses (fibrin) in arterioles caused narrowing and closure of vascular lumens (Figure 1E,F,G,H). Thrombosis was found in the arterioles, and it extended to the vascular pole of the adjacent ischemic glomerulus, without inflammatory cell infiltration (Figure 1E,F). Under electron microscopy, the glomerular capillaries contained large, intraluminal, finely vacuolated extracellular masses. The vacuoles were predominantly electron lucent, suggesting lipid “thrombi.” The subendothelial zone was focally expanded by electron-lucent material (Figure 1I,G). LPG with TMA was diagnosed. In our hospital, funduscopic exams revealed significant cotton exudates in both retinas. Serum apoE was 7.2 mg/dL. Serum ADAMTS13 and fecal Shiga toxin were not measured. Although she received fenofibrate (200 mg per day), hemodialysis, antihypertensive therapy and other supportive therapy, renal failure could not be improved.

**Figure 2:**
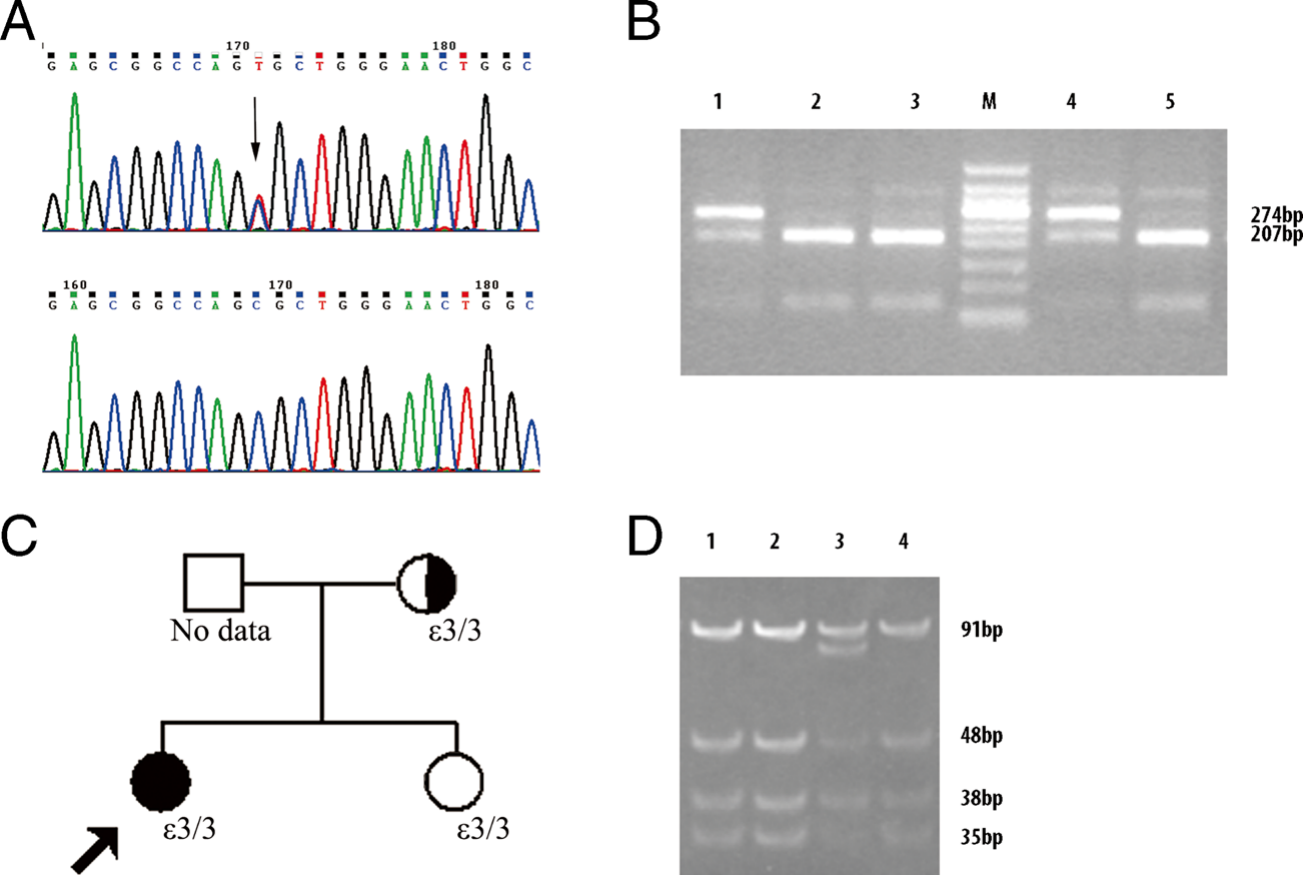
**Mutation Detection, Genotyping of ApoE and Family Pedigrees of Patients with Lipoprotein Glomerulopathy (LPG) and TMA. **Panel **A **shows DNA sequencing results of *APOE* exon 3 of the patient and a healthy control and the positions of the *APOE* Kyoto mutation (arrow). The LPG patient had a heterozygous C→T mutation leading to an amino acid substitution of Cys for Arg at codon 25 of apoE. Panel **B **shows the results of *APOE *Kyoto detection with PCR-RFLP analysis. Lanes 1 (the patient) and 4 (patient’s mother): the presence of 274 bp and 207 bp fragments indicate heterozygous mutation of *APOE *Kyoto. Lanes 2 (patient’s sister), 3 and 5 (healthy control): the presence of the 207 bp fragment indicates the wild-type homozygote. M: 100 bp molecular ladder. Panel **D **shows the results of genotyping with PCR-RFLP analysis. Lanes 1 (the patient), 2 (patient’s sister), and 4 (patient’s mother): the presence of 91 bp, 48 bp, 38 bp and 35 bp fragments indicates an ε3/3 genotype. Lanes 3 (control): the presence of 91 bp, 83 bp, 48 bp, 38 bp and 35 bp fragments indicates an ε2/3 genotype. M: pUC19 DNA/MspI marker. In Panel **C**, family pedigree for the patient indicates the *APOE *Kyoto genotype in probands (arrows) and family members from whom DNA was available. No other family members had clinical evidence of the disease. Men are represented by squares, and women by circles. The shading indicates patients with LPG. The patient’s mother is an asymptomatic *APOE *Kyoto carrier.

Genomic DNA of the patient was extracted from peripheral blood lymphocytes using DNA-isolation kits (TaKaRa, Ostu, Japan). The patient, her sister, and her mother were screened for mutations in the coding sequence of *APOE*. Four pairs of primers (see Additional file 1: Appendix Table S1) were designed with Primer Premier 5.0 to amplify all exons and adjacent intron/exon boundaries of *APOE* (reference sequence GenBank accession no. NM_000041). PCR products containing the sequences of exons 1, 2, and 3 were sequenced directly in both directions, and that of exon 4 was cloned into the pGEM-T vector (Promega, Madison, WI), and sequenced on an ABI377A DNA sequencer (Applied Biosystems, Foster City, CA). *APOE* Kyoto mutation was identified (Figure 2A). Family study (Figure 2B,C) showed that her mother also carried the *APOE* Kyoto mutation, but she did not present any evidence of renal dysfunction, proteinuria or thrombocytopenia. Restriction fragment length polymorphism (RFLP) analysis for *APOE* genotype [3] showed these *APOE* Kyoto mutations were located in ε3 (Figure 2D). Her sister did not carry *APOE* Kyoto. Written informed consents were obtained from the patient and her relatives prior to commencement of the genetic analysis.

## Conclusions

Lipoprotein glomerulopathy (LPG) is a rare inherited kidney disease, mainly caused by the *APOE* mutation. Less than 80 cases have been reported worldwide since 1989 [4], mainly among persons of Japanese and Chinese origin. Affected patients typically present with nephrotic syndrome, hypertriglyceridemia and elevated plasma apoE levels. Hypertension had been thought to be mild [5]. Approximately half of the reported cases in the literature developed renal failure 1–27 years after onset of disease [6]. Risk factors hastening the progression of LPG have not been clearly identified. On kidney biopsy, intraglomerular lipoprotein thrombi due to deposition of lipid droplets within the lumina of severely dilated glomerular capillaries are the distinct finding for this disease. However, no thrombi in arterioles have been described. TMA has not been previously reported in LPG.

Our case demonstrated LPG and TMA. The patient presented nephrotic syndrome with elevated triglyceride and cholesterol. Light microscopic examinations of renal biopsy material showed a marked dilatation of the capillary lumen in glomeruli with a mesh-like material. Electron microscopy confirmed the presence of extensive lipid thrombi in capillary lumen. LPG had been diagnosed. However, it is hard to attribute acute renal dysfunction, severe anemia and thrombocytopenia to typical LPG. Light and electron microscopic examinations of renal biopsy material showed arteriole thrombosis, endothelial swelling of arterioles, fibrin exudation in the lumina of arterioles, glomerular shrinkage and widening of the subendothelial zone. These thrombi in arterioles were different from lipo-thrombi in glomerular capillaries. The former was stained PAS positive, while the latter had a pale color with PAS and pale green with Masson’s trichrome. These thrombi in arterioles should not be the result of a direct deposition of abnormal lipoprotein. Based on elevated blood pressure, acute renal failure, elevated indirect bilirubin and LDH, thrombocytopenia and these renal pathologic findings, LPG with TMA was diagnosed, which has not been previously described in any LPG patient.

TMA is characterized by a syndrome of microangiopathic hemolytic anemia, thrombocytopenia, and variable signs of organ injury due to platelet thrombosis in the microcirculation [7]. TMA usually encompasses hemolytic uremia syndrome (HUS) and thrombotic thrombocytopenic purpura (TTP). TMA can be caused under some predisposing conditions, such as pregnancy, cancer [8], hematopoietic stem cell transplantation [9], HIV infection, systemic lupus erythematosus [10], scleroderma, cyclosporine or antineoplastic treatment [11-13] and malignant hypertension [14,15], which are classified as secondary forms of TMA. It is difficult to distinguish among these diagnoses without clinical information.

Malignant hypertension was defined as severe elevation of mean arterial pressure in combination with grade III to IV hypertensive retinopathy, according to the Keith-Wagener and Barker classification. It is often accompanied by target organ damage, such as visual disturbances, headache, heart failure, stroke or transient ischemic attack, acute renal dysfunction. TMA was observed in 27% of patients with malignant hypertension at the emergency department [16]. The pathogenesis of TMA in malignant hypertension is not precisely understood. It is postulated that the endothelial dysfunction caused by activation of the renin-angiotensin-aldosterone system (RAAS) has a central role. Reduction of NO and activation of a coagulation cascade leads to fibrinoid necrosis, edema of arterioles and local platelet aggregation. Microangiopathic hemolysis has been attributed to the mechanical stress when red blood cells pass through the narrowed lumen of arterioles. Rapid control of hypertension could ameliorate TMA and contribute to the improvement of renal function [16].

In this case, the patient suffered severe headache, blurry vision, nausea, vomiting, acute renal failure, grade III hypertensive retinopathy, and microangiopathic hemolytic anemia with blood pressure 220/160 mmHg. Thrombocytopenia, anemia and elevated LDH improved with blood pressure control. All of the above supported the patient suffered TMA which could be triggered by malignant hypertension. Preexisting renal parenchymal diseases were identified in 14% patients of malignant hypertension [16]. It is reasonable to presume that malignant hypertension was triggered by uncontrolled LPG in this patient.

Most patients with TMA due to malignant hypertension have platelet counts of over 50 × 10^9^/L and hemoglobin of over 6.5 g/dl [16-18]. In this case, the patient showed severe anemia (3.8 g/dL) and thrombocytopenia (29 × 10^9^/L), which is very rare in TMA due to malignant hypertension. As is known, there is a considerable amount of lipoprotein thrombi in intraglomerular capillaries in LPG, and blood passes through the narrow space between the capillary wall and thrombus (Figure 1). We presume that in malignant hypertension, the above mentioned lipoprotein thrombi led red blood cells to be more vulnerable to shear stress, which could aggravate microangiopathic hemolysis and worsen renal function. However, in view of severe thrombocytopenia and anemia in this patient and limited LPG cases reported, the connection between LPG and TMA may be coincidental and need further investigation.

Malignant hypertension with TMA is a medical emergency. Its outcome depends on adequate control of blood pressure and serum creatinine on presentation [19]. For this case, the control of hypertension ameliorated microangiopathic hemolytic anemia, but did not improve renal failure. For LPG patients, early renal biopsy is crucial, because an early accurate diagnosis and treatment of LPG shows remission and survival benefit. Protein A immunoadsorption [20] and LDL-apheresis [21] could induce remission of LPG, but are very expensive. Treatment with intensive lipid-lowering agents, including fibrates, has been reported to lead to clinical remission along with histological resolution of lipoprotein thrombi in serial biopsies [22-25]. Clinical remission of LPG may reduce the risks of malignant hypertension with TMA. Therefore, early biopsy, effective treatment (fibrate-containing treatment), and adequate control of blood pressure are important to improving the outcome of LPG.

Previous literature has not reported that cyclophosphamide alone with moderate dosage can induce TMA [26,27]. This patient only received cyclophosphamide with a total dosage of 1 g and had maintained stable renal function, normal hemoglobin and platelet count over the next three years. Therefore, for this patient, cyclophosphamide did not appear to be the cause of TMA three years later.

In summary, we report herein a rare case of LPG that developed severe TMA due to malignant hypertension. Adequate control of blood pressure ameliorated microangiopathic hemolytic anemia, but not renal failure. For LPG, early diagnosis and treatment may lower the risk of malignant hypertension with TMA. The factors that may trigger this course and how patients should be treated has not yet been determined. Careful observations of renal tissue under light and electronic microscopy, especially for arterioles are key to diagnosing LPG with TMA.

## Consent

Written informed consents were obtained from the patient and her relatives for publication of this case report and any accompanying images.

## Abbreviations

LPG: Lipoprotein glomerulopathy; TTP: Thrombotic thrombocytopenic purpura; HUS: Hemolytic uremic syndrome; TMA: Thrombotic microangiopathy.

## Competing interests

The authors declare that they have no competing interests.

## Authors’ contributions

YW, XHC, BHW, XXL, YT, PF, ZXH were the physicians treating the patient in this report. BHW and ZXH performed the renal biopsy evaluation and the pathology studies. YY performed genetic studies. The manuscript was prepared by YW, XHC, BHW, YY, YT, PF and ZXH. All authors participated in discussions about the manuscript and approved the final version.

## Pre-publication history

The pre-publication history for this paper can be accessed here:

http://www.biomedcentral.com/1471-2369/14/53/prepub

## Supplementary Material

Additional file 1: Appendix Table S1 Primer Sequences, Annealing Temperatures, and Product Sizes of the PCR Products. Click here for file
