# Supplementary material for: A case of lipoprotein glomerulopathy with thrombotic microangiopathy due to malignant hypertension
Source: BMC Nephrol. 2013 Feb 28;14:53. doi: 10.1186/1471-2369-14-53 (PMC3598816; doi:10.1186/1471-2369-14-53)
Supplement: Additional file 1: Appendix Table S1 — Primer Sequences, Annealing Temperatures, and Product Sizes of the PCR Products. [file 1471-2369-14-53-S1.doc]

**Supplementary Appendix Table 1**

Primer Sequences, Annealing Temperatures, and Product Sizes of the PCR Products.

| Exon | Primer sequence (5’→3’) | Annealing temperature (°C) | PCR product size (bp) | Restriction endonuclease | Digestion products (bp) |
| --- | --- | --- | --- | --- | --- |
| Exon 1 | F: GAACAGCCCACCTCGTGACT  R: GTCCCCTGCTGCTTGCCTC | 59 | 308 |  |  |
| Exon 2 | F: TAAATGTGCTGGGATTAGGCT  R: CAGGAAGCAGCACAGAAGC | 57 | 326 |  |  |
| Exon 3 | F: ACCGACTCCCCCCTCACCCT  R: CCCCCCAAGACTTAGCGACA | 57 | 424 |  |  |
| Exon 4 | F: CCTCTTGGGTCTCTCTGGCT  R: GAGAGGAGGGGGCTGAGAAT | 59 | 1056 |  |  |
| Exon 3R | F: GGAGCAAGCGGTGGAGACA  R: CCCCCCAAGACTTAGCGACA | 60 | 274 | Eco47III | Wild type: 207+67  Mutant: 274 |
